# Vector Competence for Dengue-2 Viruses Isolated from Patients with Different Disease Severity

**DOI:** 10.3390/pathogens9100859

**Published:** 2020-10-21

**Authors:** Ronald Enrique Morales-Vargas, Dorothée Missé, Irwin F. Chavez, Pattamaporn Kittayapong

**Affiliations:** 1 Department of Medical Entomology, Faculty of Tropical Medicine, Mahidol University, Bangkok 10400, Thailand; 2 MIVEGEC, University Montpellier, IRD, CNRS, 34394 Montpellier, France; dorothee.misse@ird.fr; 3 Department of Tropical Hygiene, Faculty of Tropical Medicine, Mahidol University, Bangkok 10400, Thailand; irwin.cha@mahidol.edu; 4 Center for Vectors and Vector-Borne Diseases, Faculty of Science, Mahidol University at Salaya, Nakhon Pathom 73170, Thailand; pattamaporn.kit@mahidol.ac.th; 5 Department of Biology, Faculty of Science, Mahidol University, Rama 6 Road, Bangkok 10400, Thailand

**Keywords:** dengue virus, disease severity, vector competence

## Abstract

Dynamics of dengue serotype 2 virus isolated from patients with different disease severity, namely flu-like classic dengue fever (DF) and dengue shock syndrome (DSS) were studied in its mosquito vector *Aedes aegypti*. We compared isolate infectivity and vector competence (VC) among thirty two *A. aegypti*-viral isolate pairs. Mosquito populations from high dengue incidence area exhibited overall greater VC than those from low dengue incidence area at 58.1% and 52.5%, respectively. On the other hand, the overall infection rates for the isolates ThNR2/772 (DF, 62.3%) and ThNR2/391 (DSS, 60.9%), were significantly higher than those for isolates ThNR2/406 (DF, 55.2%) and ThNR2/479 (DSS, 54.8%). These results suggest that the efficacy of dengue virus circulation was likely to vary according to the combination between the virus strains and origin of the mosquito strains, and this may have epidemiologic implications toward the incidence of flu-like classic dengue fever (DF) and dengue shock syndrome (DSS).

## 1. Introduction

Flulike classic dengue fever (DF) and dengue hemorrhagic fever (DHF) are increasingly important public health problems issues in the tropical region. Annually, an estimated 390 million dengue infections per year and 96 million of symptomatic dengue infections occur [1]. It has been predicted that population at risk of dengue will rise by 2.25 (1.27–2.80) billion by the year 2080 compared to 2015, which will put over 6.1 (4.7–6.9) billion at risk—nearly 60% of the world’s projected 2080 population [2]. DF/DHF and DSS has re-emerged in tropical and subtropical zones, with DHF as the prominent cause of pediatric mortality and hospitalization during the past 20 years in Southeast Asia [3,4]. All dengue viruses (DENV) can result to DHF cases, but the severe form is mostly associated with DENV-2 and DENV-3 [5]. It has been suggested that DHF epidemics occur as a consequence of a very complex mechanism comprising the intersection of three groups of factors (viral, host, and epidemiological) [6]. In addition, some amino acid changes on the premembrane (prM) and envelope (E) proteins of DEN-2 strains have been related to DHF epidemics [7]. Furthermore, Pandy and Igarashi classified Thailand DENV-2, according to the non-synonymous amino acid replacement, in three subtypes (I, II, and III) and proposed that viruses’ molecular structures and patients’ serological responses influence clinical severity [8].

*Aedes aegypti*, the primary vector of dengue, is well-adapted to cohabitate with humans by breeding in man-made containers. Several population genetic and vector competence (VC) studies have been done on *A. aegypti* because of its ability to transmit dengue and yellow fever flaviviruses [9,10,11,12,13,14,15,16]. Genetic markers have been used to identify biotypes, biting behavior, and other epidemiologically relevant traits [17]. Vector movement among disease foci or from a site of initial colonization can be investigated through genetic similarity between geographically heterogeneous populations [18]. The genetically intrinsic leniency of a vector to infection, replication, and virus transmission, vector competence [19], present significant variability among *A. aegypti* populations particularly for DENV-2es [10,11,20]. Genes or sets of genes controlling the midgut infection and escape barriers have been found to be associated with the VC of *A. aegypti* for flaviviruses [14,15,16].

There are several possible mechanisms that can affect the transmission potential of different dengue virus strains. Some strains may infect and replicate target cells more efficiently, sustain higher viremia in the human host, and infect more mosquitoes [21,22,23]. Studies have shown that dengue virus serotypes and strains within a serotype, between and within a genotype may vary in their ability to infect and disseminate in mosquitoes [10,11,24,25,26].

Several studies have reported variation in the oral receptivity of *A. aegypti* for DENV-2 at a regional geographic scale [27,28,29], as well as VC [30]. Furthermore, it has also been reported that DENV-2 vary according to the genotype in their efficiency to infect and disseminate in *A. aegypti* [24,25,26]. However, these studies were done using only a few mosquito collections or virus strains resulting in the evaluation of patterns of differential infection in a small number of virus–vector pairs. 

In the present study, we address the potential variation in VC and patterns of oral infection of dengue virus isolates representing distinct disease severity in sympatric DENV-2-*A. aegypti* pairs on a regional geographic scale in Thailand. In particular, eight geographically heterogeneous mosquito strains collected from areas with high and low dengue incidence were orally challenged with four virus isolates recently isolated from patients exhibiting flu-like classic dengue fever (DF) and dengue shock syndrome (DSS).

## 2. Results

**Figure 1:**
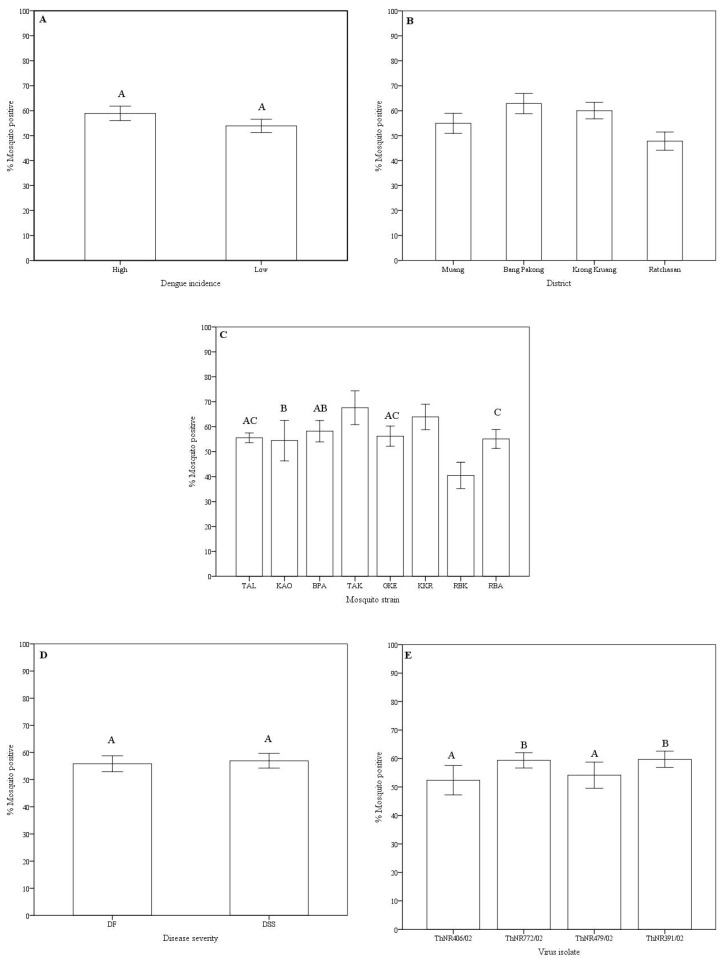
Disseminated infection rate variations of dengue virus serotype 2 isolated from patients with different disease severity in *Aedes aegypti* according to dengue incidence at the mosquito origin area (**A**), mosquito geographic origin and districts (**B**), mosquito strains (**C**), patient disease severity (**D**), and virus isolate (**E**). Mosquitoes were scored positive if RNA dengue virus was detected in head tissue by RT-PCR. Bars and error bars show the mean percentage of positive mosquitoes and the standard error, respectively. Bars with the same letter are not significantly different (*p* < 0.05), while bars without or different letter are significantly different (*p* > 0.05), based on Tukey’s test for means comparison.

Disseminated infection rates: Variations in the disseminated infection rates of the virus isolates in *Aedes aegypti* were observed according to the mosquito origin (dengue incidence at the area and location), mosquito strain, disease severity of the patients from whom the virus was isolated, and the virus isolate (Table 1 and Figure 1).

Overall, the disseminated infection rates of dengue viruses in mosquito strains ranged from 66.7% (TAK, 136 of 204 mosquitoes) to 39.2% (RBK, 98 of 250 mosquitoes). Mosquito strains collected from high dengue incidence areas showed a higher infection rate (58.1% of 854 mosquitoes) compared to mosquitoes from low dengue incidence area (52.5% of 953 mosquitoes). However, no significant statistical difference was observed (Figure 1A). Significant differences in infection rates were observed among geographic origin. Mosquitoes collected from Bang Pakong district exhibited the highest infection rate (62.1% of 422 mosquitoes) while mosquitoes collected from Ratchasan district exhibited the lowest disseminated infection rate (46.9% of 484 mosquitoes). Mosquito strains collected from the Muang district, despite being the most urbanized and with the highest reported dengue incidence, showed lower disseminated infection rate (54.9% of 432 mosquitoes) compared to mosquito strains from the rural Krong Kruang district (59.5% of 459 mosquitoes) that has lower reported dengue incidence (Table 2 and Figure 1B). Among mosquito strains, significantly different disseminated infection rates were observed, except for the BPA-GKE, BPA-TAL, BPA-KAO, GKE-RBA, and GKE-TAL pairs of mosquito strains (Figure 1C). For virus isolates grouped according to the disease severity of the patients from whom they were isolated, no differences in disseminated infection rate was observed between DF and DSS virus isolates (Figure 1D). However, disseminated infection rates of the isolates ThNR2/772 (from DF patient) and ThNR2/391 (from DSS patient), were overall significantly different from isolates ThNR2/406 (DF) and ThNR2/479 (DSS) with *p* < 0.005 (Figure 1E).

Vector competence variation: As a proxy of vector competence (VC), the VC was calculated as the number of mosquitoes positive for the viral RNA in head tissues divided by the number of mosquitoes exposed to the respective viruses. To better understand the geographical variation in the *A. aegypti* vector competence for dengue viruses isolated from patients exhibiting different disease severity (DF and DSS), the mosquito strains were grouped by dengue incidence at the area of mosquito origin (sub-district level). High dengue incidence sub-districts comprises TAL, KAO, BPA, and TAK strains, while low incidence sub-districts were KKR, GKE, RBK, and RBA strains. Mosquito strains were further classified location at the district administrative level Muang (TAL and KAO), Bang Pakong (BPA and TAK), Krong Kruang (KKR and GKE) and Ratchasan (RBK and RBA). 

**Figure 2:**
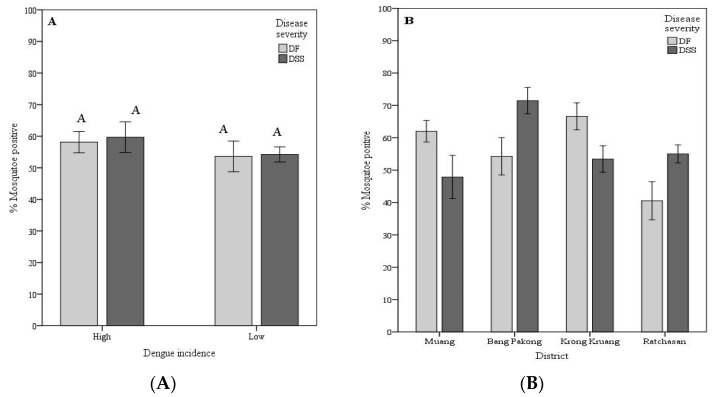

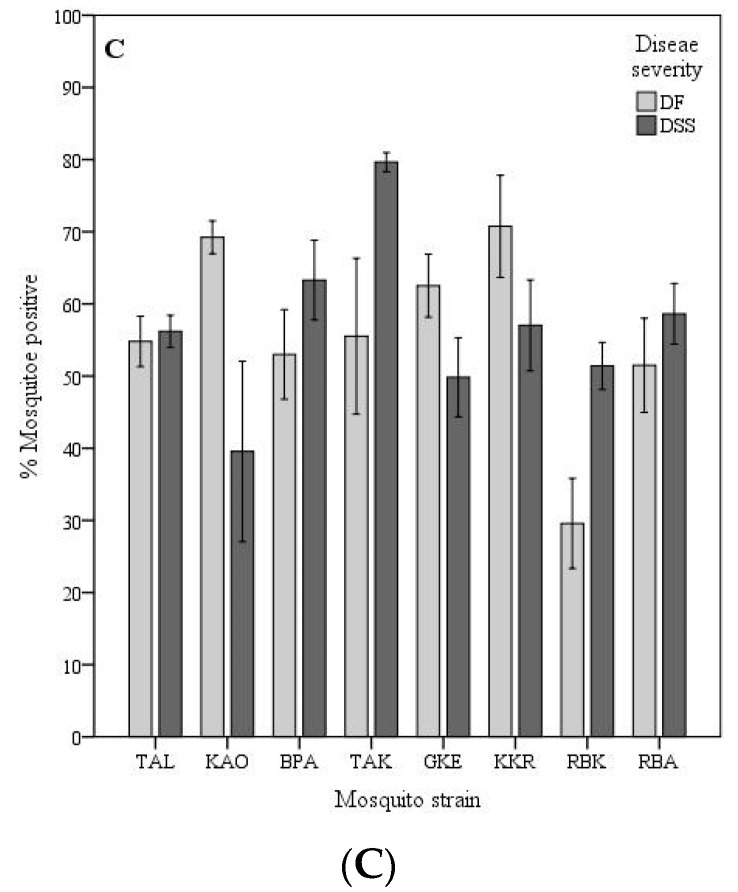
Vector competence of *Aedes aegypti* for dengue serotype 2 virus isolated from patients exhibiting different disease severity according to dengue incidence at the mosquito strain origin area (**A**), mosquito geographic origin, districts (**B**), and mosquito strains (**C**). Vector competence is expressed as the number of mosquitoes positive for viral RNA in head tissues/number tested. Bars and error bars show the mean percentage of positive mosquitoes and the standard error, respectively. Bars with the same letter within the same group are not significantly different (*p* < 0.05) based on Tukey’s test for means comparison.

For the first grouping, the VC among mosquitoes from high dengue incidence for DSS isolates ranged from 19.6% (KAO mosquito strain for the ThNR2/479 virus isolate) to 85.4% (TAK for the ThNR2/479 virus isolate). DF isolates ranged from 37.0% (TAK mosquito strain for the ThNR2/406 virus isolate) to 73.1% (TAK mosquito strain for the ThNR2/772 virus isolate). Although no statistically significant differences were observed between the overall VC for DF and DSS, mosquito strains collected from high dengue incidence areas showed an overall higher competence for viruses isolated from DSS patients than from DF patients. Whereas mosquito strains collected from low dengue incidence areas exhibited a similar overall VC for either DF or DSS virus isolates, with VC ranging from 40.6% (GKE mosquito strain for the ThNR2/391 virus isolate) to 67.3% (KKR mosquito strain for the ThNR2/391) for DSS isolates, and 18.8% (RBK mosquito strain for the ThNR2/406) to 81.8% (KKR mosquito strain for the ThNR2/406) for DF isolates (Table 1). For the second grouping, mosquito strains from Bang Pakong and Ratchasan districts showed a significantly higher VC for viruses isolated from patients with DSS compared to patients with DF symptoms (Figure 2B). The VC for mosquito strains from Bang Pakong ranged from 53.7% (for ThNR2/391 isolate virus) to 85.4% (ThNR2/479 isolate virus) for DSS isolates, whereas DF isolates ranged from 37.0% (for ThNR2/406 isolate virus) to 73.1% (ThNR2/772). The VC for mosquito strains from Ratchasan ranged from 47.2% (for ThNR2/479) to 65.5% (for ThNR2/391) for DSS isolates, and from 18.8% (for ThNR2/406) to 61.7% (ThNR2/772) for DF isolates (Table 1). In contrast, mosquito strains from Muang and Krong Kruang districts showed a significantly higher VC for viruses isolated from patients with DF than from those with DSS, (Figure 2B). The VC for mosquito strains from Muang ranged from 52.3% (for ThNR2/772) to 70.4% (ThNR2/406) for DF isolates and from 19.6% (for ThNR2/479) to 61.4% (for ThNR2/391) DSS. The VC for mosquito strains from Krong Kruang ranged from 56.2% (for ThNR2/772) to 81.8% (for ThNR2/406 isolate virus) for DF, and from 40.6% (for ThNR2/391) to 67.3% (for ThNR2/391) for DSS isolates (Table 1).

The VC of individual mosquito strains significantly differed between virus isolated from patients with DSS and DF, *p* < 0.05. Females of the TAL, KAO, GKE, and KKR strains exhibited a significantly higher VC for DF virus isolates than for DSS, while BPA, TAK, and RBK showed a significantly higher VC for DSS than for DF virus isolates, (*p* < 0.05) as shown on Figure 2C. As summarized on Table 1, the VC among mosquito strains for the four isolates differed according to the *A. aegypti* strain-virus isolate combinations which ranged from 18.8% (RBK-ThNR2/406) to 85.4% (TAK-ThNR2/479).

Viral isolates infectivity variations: The infectivity of a virus isolate for *A. aegypti* was calculated as the number mosquitoes positive for viral RNA in the abdomen divided by the number exposed to the virus isolate. Overall, ThNR2/772 (DF) and ThNR2/391 (DSS) were the most infectious virus isolates, where 62.3% of 467 and 60.9% of 422 mosquitoes were positive, respectively; while ThNR2/406 (DF) and ThNR2/479 (DSS) were significantly less infectious where 55.2% of 458 and 54.8% of 449 mosquitoes were positive, respectively, (*p* < 0.05; Figure 1E).

Different patterns of infection dynamics among individual virus isolates in mosquitoes were observed. Infection rates for ThNR2/772 (DF) and ThNR2/391 (DSS) isolates were more uniform. ThNR2/772 ranged from 40% (RBK mosquito strain) to 73.1% (KAO mosquito strain), while ThNR2/391 ranged from 40% (GKE mosquito strain) to 80% (TAK mosquito strain). In contrast, virus isolates ThNR2/406 (DF) and ThNR2/479 (DSS), exhibited more phenotypic variation where infection rates ranged from 18.8% (RBK mosquito strain) to 88.6% (KKR mosquito strain), and from 21.6 (KAO mosquito strain) to 85.4% (TAK mosquito strain), respectively, (Table 1). Furthermore, according to the mosquito strains, significant differences in the number of infected mosquitoes were observed among individual viral isolates. The proportion of mosquitoes infected by the ThNR2/406 (DF) virus isolate was not significantly different only between KAO-GKE and TAK-RBA mosquito strain pairs, and by the ThNR2/772 (DF) virus isolate was not significantly different between GKE-KKR-RBA mosquito strains, and by the ThNR2/391 (DSS) virus isolate was not significantly different between TAL-KAO-BPA-RBK mosquito strains, and by the ThNR2/479 (DSS) viral isolate was not significantly different only between KKR-RBA mosquito strains.

## 3. Discussion

Vector, viral, and environmental factors determine the capacity of dengue virus to infect and disseminate in its mosquito vector. In the present study, we challenged each mosquito strain with all four virus isolates at the same time and mosquitoes were held together during the same environmental fluctuations of the incubation period (14 days), to minimize the effect of environmental variation. Even though all mosquito strains may not have experienced the same environmental conditions no statistical significant differences were observed when mean temperature and humidity that each mosquito strain experienced during the incubation period were compared among strains (data not shown). Nonetheless, some of the observed mosquito VC and viral isolate infectivity variations may have been influenced by environmental variation among experimental groups.

Variations in infection rates of *A. aegypti* experimentally infected with DEN virus has been reported [10,11,20,27,28,29,30]. Differences on the proportion of infected mosquitoes between DENV-2 with SEA and American genotype have also been reported in geographically separated *A. aegypti* populations [24,25,26]. In contrast to previous studies, the present study used low-passage virus isolates that were recently isolated from patients exhibiting different disease severity (i.e., DF and DSS), which were of sympatric geographic origin with the used *A. aegypti* strains. Although the *A. aegypti* were collected in the same province, considerable variability in overall infection rates was observed according to the dengue incidence in the collection area, geographic origin (districts), mosquito strain, individual virus isolate, and disease severity of the patients from whom the virus was isolated (Figure 1A–E). 

From our observations, although neither high or low dengue incidence mosquito strains showed significantly different VC for DF or DSS viral isolates (Figure 2A), the mosquito strains collected from high dengue incidence sites exhibited higher overall VC for virus isolates either isolated from patients exhibiting mild (DF) or severe (DSS) disease severity than mosquito strains from sites with low dengue incidence site (Table 3). However, significant differences in VC for DF and DSS virus isolates were observed among mosquito strains from different geographic origins (districts) (Figure 2B). Furthermore, most individual mosquito strains showed significantly different VC either for DF or DSS virus isolates. Only the RBA mosquito strain did not show significantly different VC between DF and DSS viral isolates (Figure 2C). Therefore, we speculate that factors such as the origins of mosquitoes, the viral strains (DF or DSS) circulating in a given locale, and mosquito factors, per se play an important role in determining *A. aegypti* VC. A detailed genetic analysis is needed to determine if these mosquito strains are actually isolated and constitute independent populations that differ in susceptibility to the infection with dengue virus. In previous studies, it has been shown that mosquitoes from populated and urbanized areas are genetically highly differentiated and exhibit high and heterogeneous infection rates, and this genetic differentiation has been related to the intensity of insecticide control and human population density [16,27,28,29]. As for mosquito factors, the VC for arboviruses is associated with anatomic barriers to productive vector infection, including a midgut infection barrier (MIB), a midgut escape barrier (MEB), and a salivary gland barrier [30,31]. *A. aegypti* VC for DENV-2 is considered as being universally proportional to the level of MIB and MEB found in mosquito collections, where strong MIBs and MEBs decrease transmission potential [30]. It is also important to remember that there is potential for different loci to affect VC in different populations [14,16]. Thus, the need to look for genetic variation is evident. This study also raised the possibility that VC for a virus isolate is not randomly distributed across the disease severity of the patient from whom the virus was isolated. We found that mosquito strains from the same district exhibited the same VC pattern, namely TAL, KAO, and GKE, KKR mosquito strains collected from Muang and Krong Kruang districts, respectively, were significantly more competent for DF virus isolates than for DSS viral isolates. In addition, the BPA and TAK mosquito strains collected from Bang Pakong districts and RBK and RBA from Rachasan district, though RBA did not show significant different VC, exhibited the same VC pattern, indeed, were more competent for DSS than for DF viral isolate (Figure 2C). 

**Figure 3:**
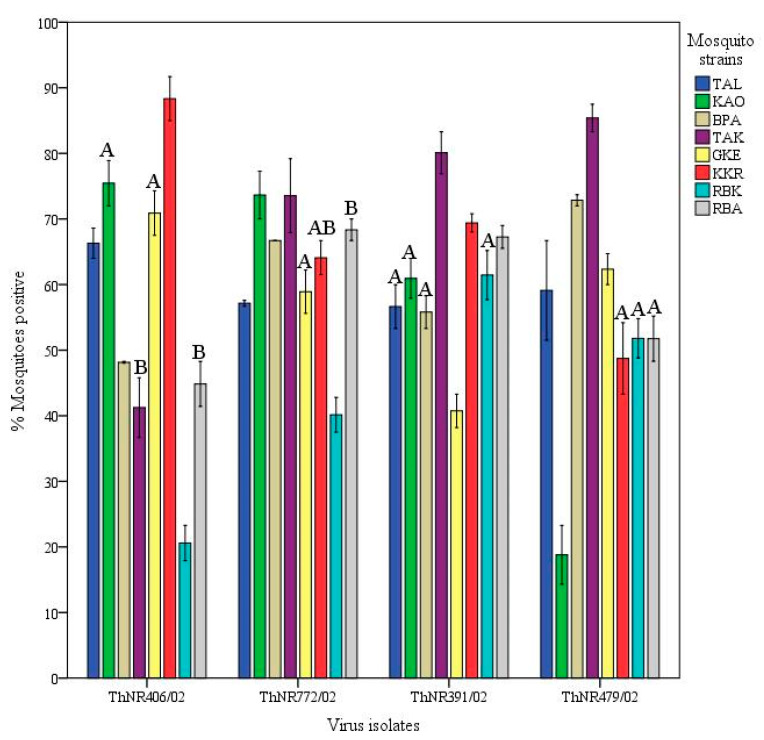
Infectivity of four dengue 2 viruses isolated from patients exhibiting different disease severity for geographically different *Aedes aegypti* strains. Virus infectivity is expressed as the number of mosquitoes positive for viral RNA in abdomen/number tested. Bars and error bars show the mean percentage of positive mosquitoes and the standard error, respectively. Bars with the same letter within the same virus isolate are not significantly different (*p* < 0.05), while bars without or different letter are significantly different (*p* > 0.05), based on Tukey’s test for means comparison.

Dengue viruses’ efficiency to infect and disseminate in their vector mosquitoes can vary greatly. In the present study, virus infectivity was evaluated by measuring the proportion of mosquitoes infected by individual virus isolates. Overall, we found variation in the proportion of mosquitoes infected with individual virus isolates. Specifically, the ThNR2/772 and ThNR2/391 isolates were more infective than the ThNR2/406 and ThNR2/479 viral isolates (Figure 1E). However, the virus isolate infectivity seems likely to be differentiated according to mosquito strain–viral isolate pairs, Figure 3. Therefore, we speculate that differences in the proportions of infected mosquitoes among individual virus isolate are probably due to certain genetic elements of the isolated virus. Few genetic differences among dengue virus isolates may have a significant effect on their infection, dissemination and transmission by vector mosquitoes. Previous studies on DENV-2 revealed differences in the proportions of mosquitoes infected by individual virus isolates and have been associated with molecular differences among virus isolates [24,25,26,32].

## 4. Materials and Methods 

**Figure 4:**
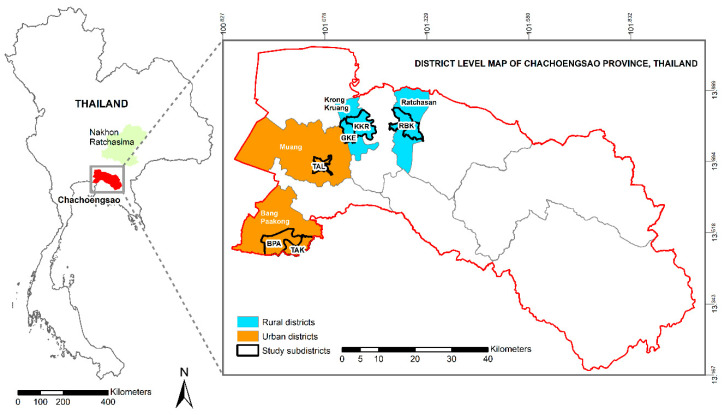
Geographic localization of the collection sites of *Aedes aegypti* used depicting the habitat type corresponding to each district.

Collection sites: Four districts in Chachoengsao province situated in eastern Thailand, with the highest (Muang and Bang Pakong) and lowest (Krong Kruang and Ratchasan) dengue (DF/DHF/DSS) incidence (per 100,000 population) reported during the past 20 years were selected. Within these districts the sub-districts (tambon) with the highest (Na Muang, Bang Pakong, and Tha Kam) and lowest (Krong Kruang, Gon Kheo, and Bang Kla) dengue incidence from 1999 to 2002 were selected as mosquito collection sites (Figure 4). Sub-districts were classified as high and low dengue risk areas based on annual incidence of DHF/DSS (cases/100,000 habitants), and the number of houses and inhabitants per square kilometer. The selected locations covered a wide geographical distribution of *A. aegypti*, including urban and rural environments. The shortest distance between study areas (districts) was ca. 20 km, and between sub-districts was ca. more than 30 km.

Mosquito collections: *A. aegypti* were collected from two sites in each sub-district, (Table 2). Samples from high dengue incidence areas were collected from houses with recent report of dengue cases and from low incidence areas in house without dengue cases for the last two years. The mosquitoes were collected as fourth stage larvae and/or pupae, at least 200 per collection site, from water containers found outside and inside the house within a radius of ca. 2 km, at collection sites separated by at least ca.5 km. Field collected mosquitoes (F0 generation) were reared to adults under room conditions (26 °C ± 2 °C, RH 75% ± 10%). After morphological identification, based on the illustrated key to the mosquitoes of Thailand [33], adults were kept in cages and females were blood-engorged on mice to produce eggs. The onward generations of mosquitoes were reared in plastic pans (33 cm × 25 cm × 11 cm) containing 3 L of aged tap water with a density of 180–210 larvae per container. The larvae were fed on a diet of mouse food powder and maintained at 26 °C ± 2 °C, RH 75% ± 10%. Adults were fed on 3% sucrose soaked cotton and females were allowed to mate and then blood-feed on mice to enable egg production. The F0 mosquitoes were stored at −80 °C for future genetic analysis. Oral infection experiments were performed with females from the first (F1) or second (F2) generations generated from the F0 proved to be free of dengue virus infection. The mosquito strains used for oral infection experiments and the dengue background of the district where the mosquitoes were collected are given in Table 2 and Figure 4. 

Virus strains: The four virus isolates were obtained from the sera of patients from Nakorn Ratchasima Provincial Hospital, Northeastern Thailand, diagnosed with DF and DSS in 2002. Clinical diagnosis confirmation and serological assays (ELISA IgG and IgM) were performed at the hospital, while virus isolation and serotype determination were performed by the staff of the Arbovirus Section, National Institute of Health, Medical Science Center, Nonthaburi. The clinical diagnosis and clinical severity gradings of each isolate was classified using the World Health Organization criteria (WHO, 1986) [34]. The serotype was determined as dengue virus type 2 by reverse transcription-polymerase chain reaction (RT-PCR). Relevant patient information is summarized in Table 3.

All isolates were passaged four times in *Aedes albopictus* clone C6/36 cell line from the patient serum before using for experiments. To ensure that the virus specimens were not altered significantly from their wild type character as found in the host patient, none of the isolate has been purified by any methodology before inoculation into mosquito cells.

Plaque assay: A seed virus was prepared by inoculation into a monolayer culture of *Ae. albopictus* clone C6/36 cell line and incubated at 28 °C for 8 days in Eagle’s medium supplemented with 2% heat-inactivated fetal bovine serum (FBS) and 0.2 mM each of nonessential amino acids plus antibiotics (20 µg/mL Gentamicin, 5 µg/mL Amphotericin B, 200 U/mL). The infected culture fluid was harvested 8 days after inoculation, aliquoted, and stored at −80 °C until used. The virus titer of the isolates was measured by the focus formation test by using BHK-21 cells on 96-well plates [35]. The virus isolates were then used for preparation of the infectious meal, which was a blood virus sucrose solution (BVS).

Oral Infection: Only 4 to 5 day-old female mosquitoes were used to minimize age factors effects. About 60 females were placed in cylindrical pint cardboard cages covered at one end with fine non-wettable nylon mesh. These females were deprived of food for 36–40 h prior to the infectious meal, then were allowed to feed on the infectious meal (BVS) consisting of equal volumes of isolate virus suspension, washed rabbit erythrocytes, and 10% sucrose solution (as a source of energy). Drops of the infectious meal were placed on the mesh covering the cardboard cage containing mosquitoes as previously described [11,25]. Feeding time was limited to 1 h, mosquitoes were cold anesthetized and fully engorged mosquitoes were collected with an aspirator at 30 min intervals, transferred into clean carton cups and maintained for up to 14 days at 30 ± 1 °C as a virus extrinsic incubation period (EIP). After the EIP completed survived mosquitoes were frozen at −80 °C, then transfer individually into 1.5 mL tube and kept at −80 °C until further use for viral RNA detection. Mosquito strains were orally challenged on different days; however, each strain was challenged simultaneously with the four virus isolates on the same day. All feeding suspensions contained the same virus titer (1 × 10^2^ PFU/mL) with an aliquot of the same virus pool. The titers of the post feeding virus suspensions did not change significantly.

Detection of virus infection: After has completed the EIP (14 days) mosquito head and abdomen were severed and homogenized in Trizol LS reagent for total RNA extraction, and then assayed for dengue virus genome by using RT-PCR method. The detection of viral RNA in the homogenized abdomen was interpreted as indicating that the mosquito midgut had become infected. Detection of RNA viral in the homogenized head indicated disseminated from the infected midgut to a secondary target organs.

RNA extraction and RT-PCR: Total RNA was extracted from individual mosquito head and abdomen severed using Trizol LS (Invitrogen) according to the manufacturer’s recommendations. Briefly, the head and abdomen of mosquito were separately homogenized in 300 µL of Trizol reagent then 99.9 µL of chloroform was added. The resulting mixed suspension was centrifuged at 14,000 rpm/min for 10 min at 4 °C. RNA was precipitated from the aqueous phase (ca. 145 µL) by mixing with 133.2 µL of isopropyl alcohol. Isopropyl alcohol-RNA precipitate was recovered by centrifugation and the RNA pellet was washed once with 90 µL of 75% ethanol followed by a second wash with absolute ethanol and then air dried. The RNA pellet was resuspended in 10 µL of RNase-free water and used as a template RNA in reverse transcriptase PCR (RT-PCR). Synthetic oligonucleotide primer pairs were designed based on published sequence data for dengue virus serotype specific primer, sense (D2-S) and complementary (D2-C) genome nucleotide (nt) regions 1203 to 1222 and 1432 to 1413 respectively [36,37].

Dengue virus RNA was assayed by using one step RT-PCR kit (Quiagen) following the manufacturer’s recommendations with slight modifications. Briefly, 2 µL of RNA, 8 µL of reaction mix, and RNase free water was reverse transcribed and amplified following the thermal cycle protocol recommended in the kit instruction manual, with some modifications, as follows: one cycle at 53 °C for 30 min, one cycle at 94 °C for 2 min, followed by 45 cycles at 94 °C for minute, 53 °C for 1 min, and 68 °C for 2 min, followed by one cycle at 68 °C for 7 min. The reaction mixture solution contained a mixture of 5 µL of 5× buffer (contains 12.5 mM MgCl_2_), 1 µL of dNTP mix (containing 10 mM of each dNTP), 0.5 µL (25 pmol) of corresponding primers, D2-S (5′-GTTCGTCTGCAAACACTCCA-3′) and D2-C (5′-GTGTTATTTTGATTTCCTTG-3′), and 1 µL of enzyme mix (an optimized combination of Omnscript Reverse Transciptase, Sensiscript Reverse Transcriptase, and HotStarttTap DNA Polymerase). Nine microliters of PCR product was subjected to agarose gel electrophoresis, and amplified DNA fragments were visualized with ethidium bromide staining.

Statistical analysis: One-way analysis of variance was used to test the variation among disseminated infection rates, vector competence measures, and virus infectivity rates. Tukey’s HSD was used for pairwise comparisons. Analogous non-parametric test were used when departures from normality or equality of variance were encountered. Statistical Package for the Social Sciences (SPSS) 11.5 software package was used to generate all the results and graphs in this study.

## 5. Conclusions

In this study, we analyzed 32 recently collected vector–virus pairs (eight geographically different mosquito strains versus four isolate virus strains). Among vector–virus pairs infection rates were different; indeed, many of those were significantly different. This leads us to conclude that the observed variations in susceptibility among mosquito strains is probably due to the combination of certain genetic elements of the vector and the virus as has been previously observed in similar researches. Furthermore, these results suggest that the efficacy of dengue virus circulation in a given locale may vary according to the interaction of virus strains with the origin of the vector mosquitoes. This study contributes to the knowledge of the role of the association between mosquito genetic determinants of susceptibility and genetic variation of dengue virus in the occurrence of different disease severity.

## Figures and Tables

**Table 1:** Infection and dissemination rates (%) ^A^ of DENV-2es isolated from patients with different disease severity in orally challenged *Aedes aegypti* strains.

Mosquito Strains	ThNR2/406 (DF) ^B^		ThNR2/772 (DF)		ThNR2/391 (DSS)		ThNR2/479 (DSS)	
	Infec	Disse	Infec	Disse	Infec	Disse	Infec	Disse
TAL	66.7	58.3 (60) ^C^	57.1	52.4 (63)	56.4	56.4 (55)	55.5	52.4 (63)
KAO	75.0	70.4 (44)	73.1	67.3 (52)	61.3	61.4 (44)	21.6	19.6 (51)
BPA	48.1	42.6 (54)	66.7	63.6 (66)	55.5	53.7 (54)	72.7	72.7 (44)
TAK	40.7	37.0 (54)	73.1	73.1 (52)	80.0	78.0 (50)	85.4	85.4 (48)
KKR	88.6	81.8 (44)	64.0	60.0 (50)	70.1	67.3 (48)	48.1	46.3 (54)
GKE	70.7	69.3 (75)	59.4	56.3 (64)	40.6	40.6 (64)	62.7	59.3 (59)
RBK	18.8	18.8 (69)	40.0	40.0 (60)	61.2	55.1 (49)	51.4	47.2 (72)
RBA	44.8	41.4 (58)	68.3	61.7 (60)	67.2	65.5 (58)	51.7	51.7 (58)

^A^ Infection and dissemination: number of mosquitoes for viral RNA in abdomen and head tissue, respectively; ^B^ DF: dengue fever; DSS: dengue shock syndrome; ^C^ Total number of mosquitoes tested for each mosquito/virus combination.

**Table 2:** Demographic information and dengue (DEN) background of the origin of *Aedes aegypti* strains from Chachoengsao province, central eastern Thailand, used in this study.

Strain	At Collection Site			At Collection Area		
	Subdistrict	Demographic ^A^	Incidence ^B^	District	Demographic ^A^	Incidence ^B^
TAL	Na Muang	4480.9/1640.4	153.2	Muang	369.2/106.8	141.3
KAO						
BPA	Bang Paakong	207.4/89.5	284.9	Bang Pakong	306.1/89.6	105.8
TAK	Tha Kam	542.5/345.2	204.2			
KKR	Krong Kruang	114.2/30.9	117.3	Krong Kruang	106.5/25.9	91.7
GKE	Gon Kheo	99.7/21.2	88.45			
RBK	Bang Kla	70.4/18.3	40.3	Ratchasan	92.4/23.4	72.1
RBA						

^A^ Numbers of people/houses per square Km. Based on census of 2002, Public Health Office, Chachoengsao; ^B^ Dengue (DHF + DSS) annual average incidence, cases/100,000 habitants. Public Health Office, Chachoengsao, 1999–2002.

**Table 3:** Medical data and in vitro infectivity results of four DEN-2 isolates virus isolated patients of Nakorn Ratchasima provincial hospital from Nakorn Ratchasima province, northeast Thailand.

Isolate Name	Medical Data						Infectivity LLC-MK2 (PFU/mL)
	Sex	Age	Clinical Diagnosis	Antibody Response	ELISA Assay (Unit)		
					Den-IgG	Den-IgM	
ThNR02/406	M	16	DF	NA ^A^	7	0	1.2 × 10^5^
ThNR02/772	M	14	DF	NA	14	9	8.5 × 10^3^
ThNR02/391	M	12	DSS	NA	29	2	1.38 × 10^5^
ThNR02/479	M	12	DSS	Secondary	3	0	1.0 × 10^2^
					149 ^B^	56	

^A^ Due to the lack of a second serum sample; ^B^ Second serum sample was obtained 2 weeks after the first sample.
